# Supplementary material for: Liquid-Crystal and Fire-Retardant Properties of New Hexasubstituted Cyclotriphosphazene Compounds with Two Schiff Base Linking Units
Source: Molecules. 2020 May 1;25(9):2122. doi: 10.3390/molecules25092122 (PMC7248761; doi:10.3390/molecules25092122)
Supplement: Supplementary file 1 [file molecules-25-02122-s001.pdf]

Supplementary Materials

Article

Liquid-Crystal and Fire-Retardant Properties of New Hexasubstituted Cyclotriphosphazene Compounds with Two Schiff Base Linking Units

Zuhair Jamain [1,2,*], Melati Khairuddean [2,*] and Tay Guan-Seng [3]

[1] Faculty of Science and Natural Resources, Universiti Malaysia Sabah (UMS), Kota Kinabalu 88400, Malaysia
[2] School of Chemical Sciences, Universiti Sains Malaysia (USM), Penang 11800, Malaysia
[3] School of Industrial Technology, Universiti Sains Malaysia (USM), Penang 11800, Malaysia; taygs@usm.my
* Correspondence: zuhairjamain@ums.edu.my (Z.J.); melati@usm.my (M.K.)

Supplementary Materials–DSC themogram of compounds 4a-i

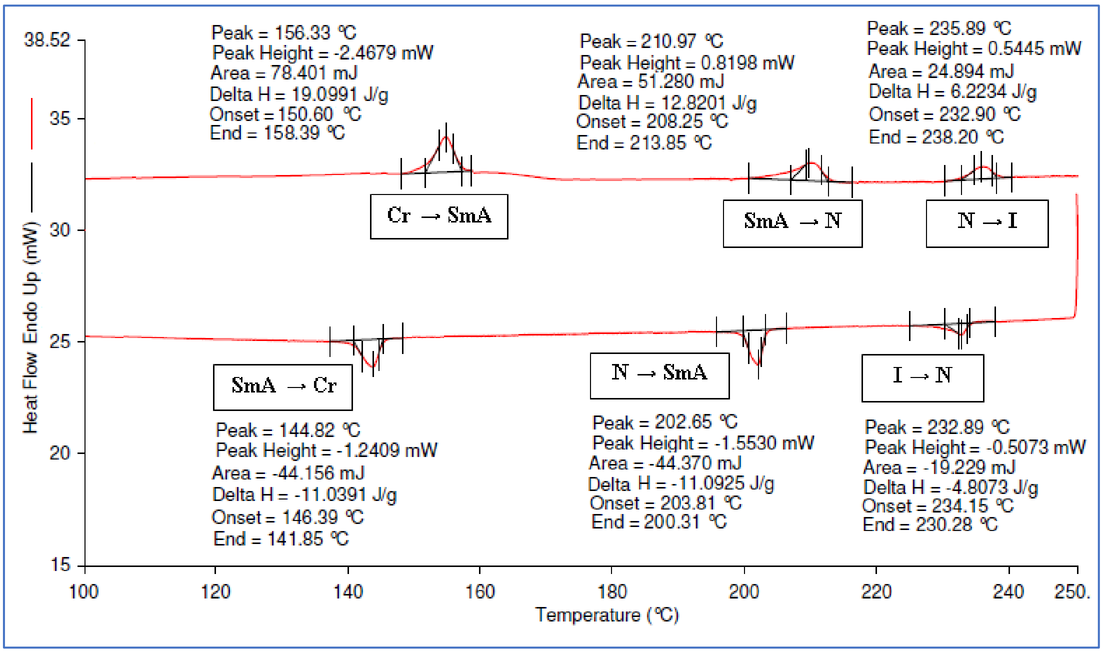

Figure S1. DSC thermogram of compound **4a**

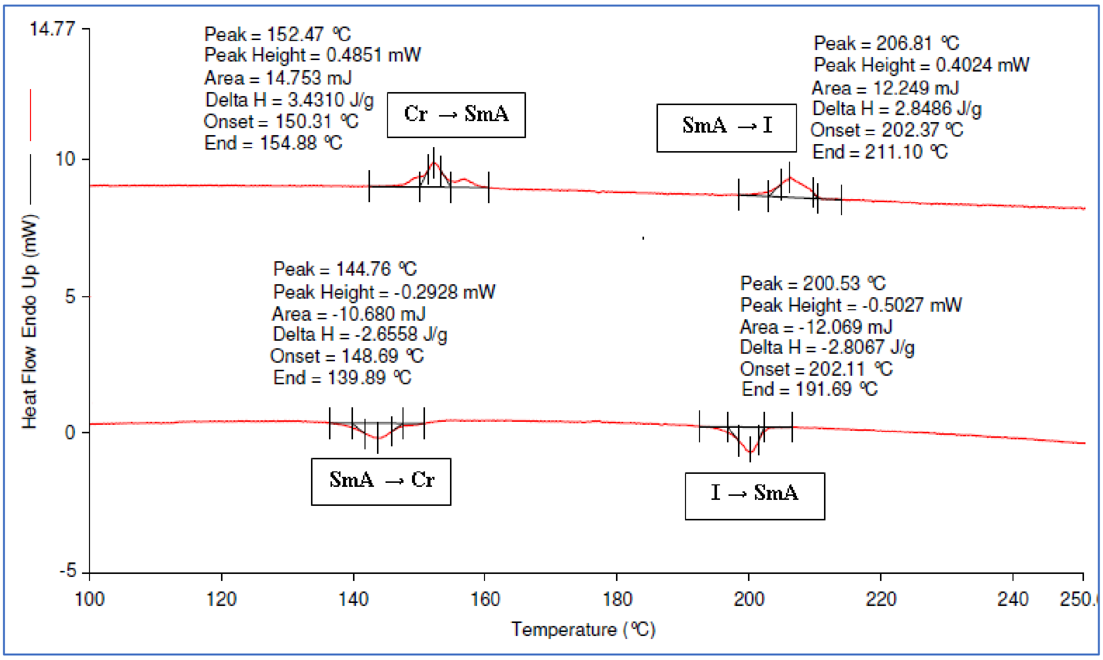

Figure S2. DSC thermogram of compound **4b**

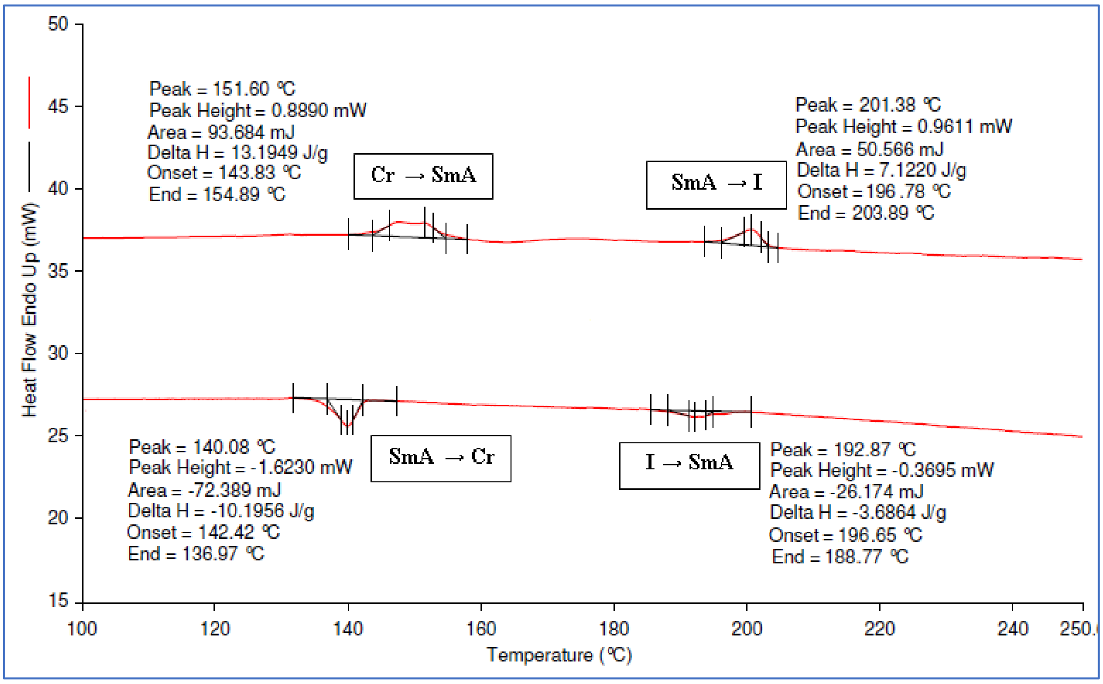

Figure S3. DSC thermogram of compound **4c**

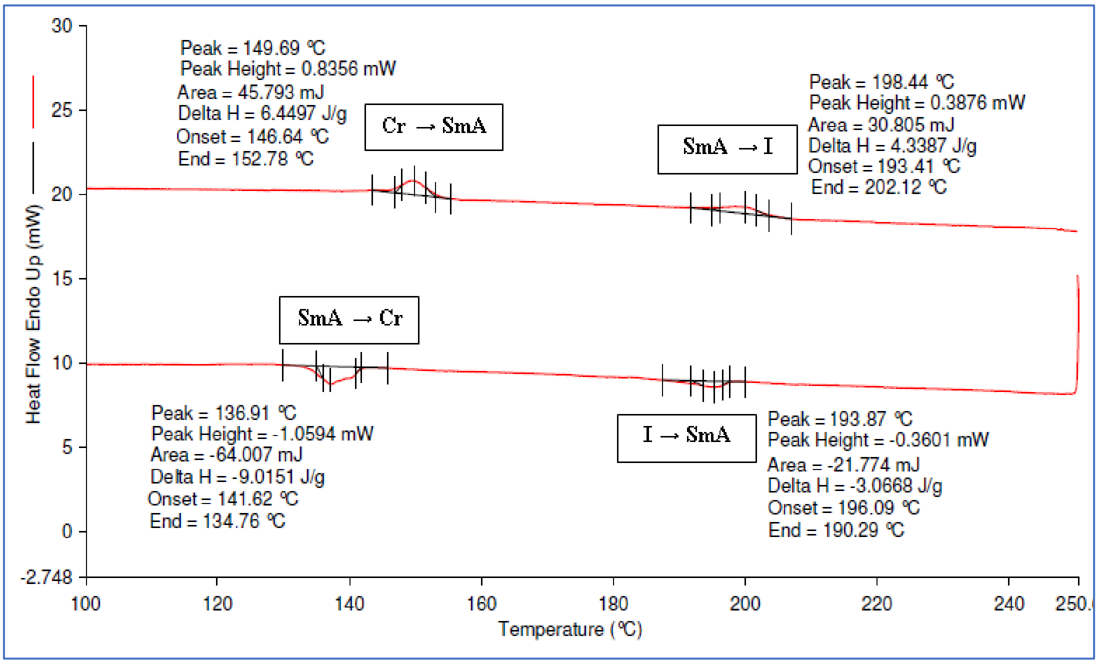

Figure S4. DSC thermogram of compound **4d**

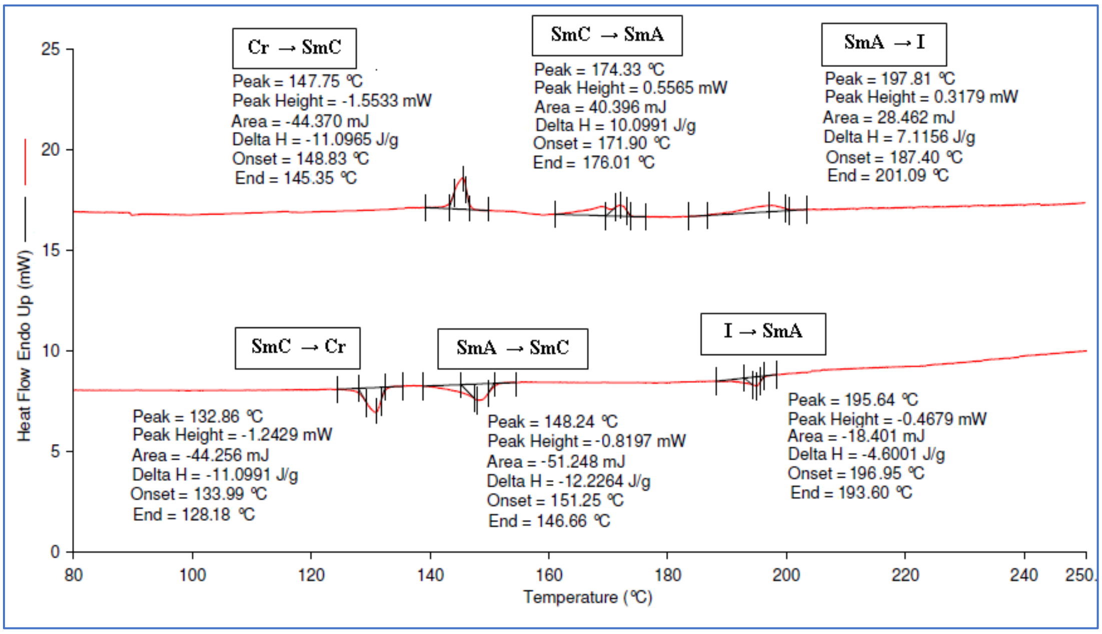

Figure S5. DSC thermogram of compound **4e**

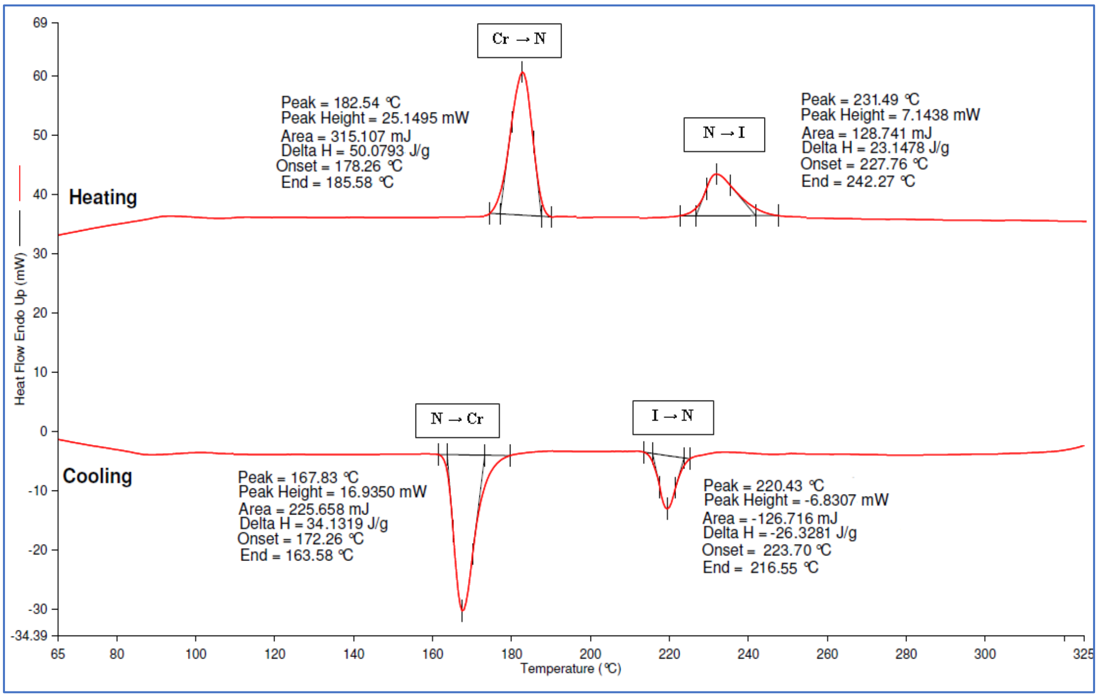

Figure S6. DSC thermogram of compound **4f**

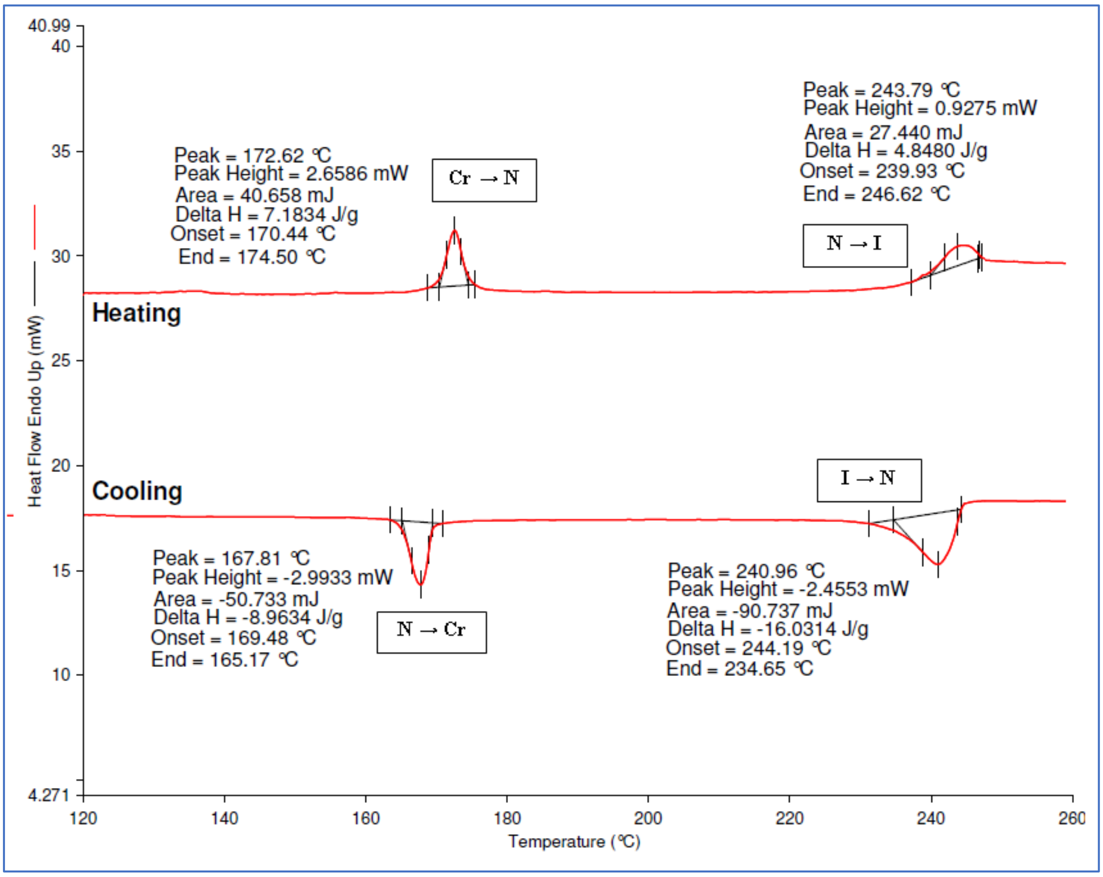

Figure S7. DSC thermogram of compound **4g**

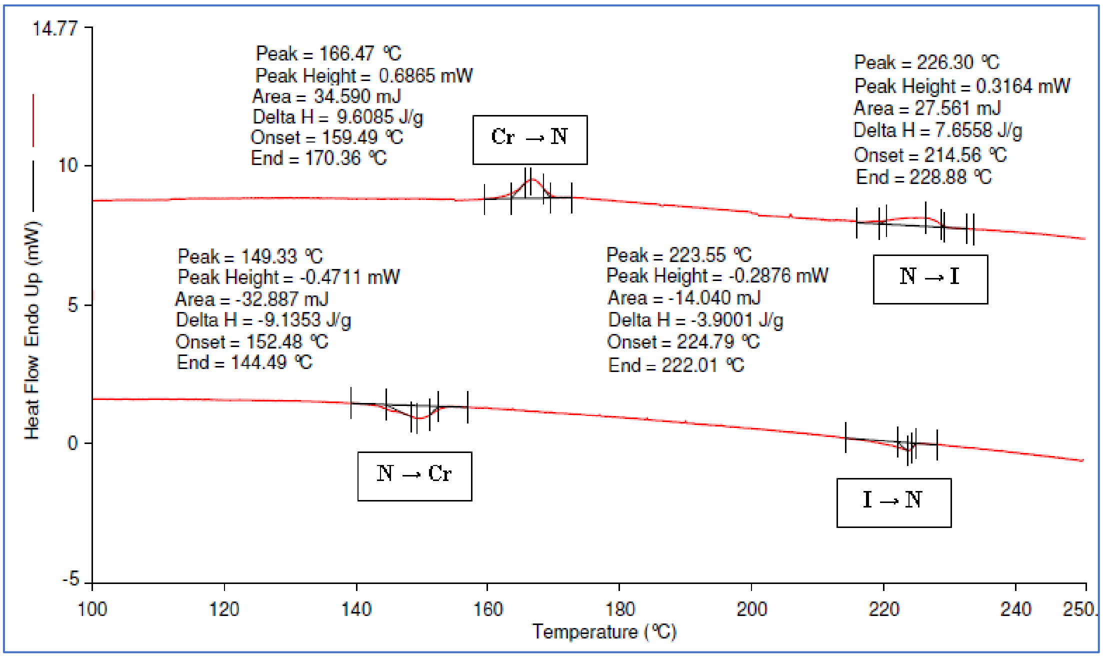

Figure S8. DSC thermogram of compound **4h**

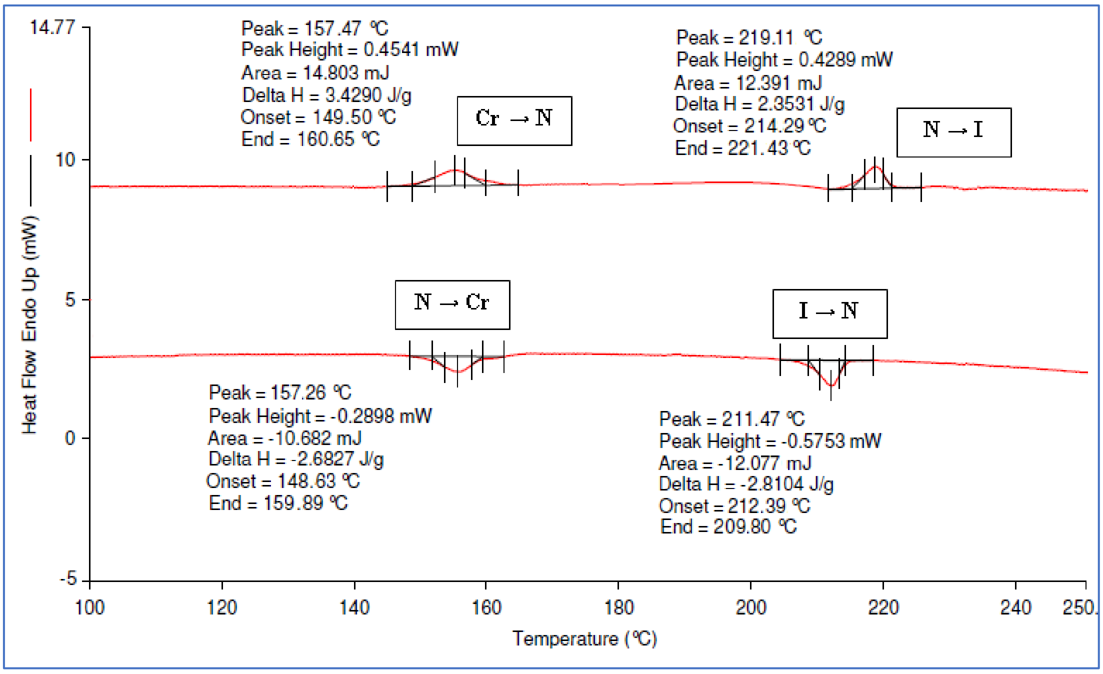

Figure S9. DSC thermogram of compound **4i**
